# Clinical evaluation of an allergen Challenge Theatre^TM^

**DOI:** 10.1186/1710-1492-10-S2-A21

**Published:** 2014-12-18

**Authors:** Jacob Karsh, Suzanne Kelly, Jimmy Yang, Rob Perrins, William H Yang

**Affiliations:** 1 Red Maple Trials Inc., Ottawa, Ontario, Canada

## Background

Allergen challenge chambers expose allergen-sensitive subjects to a predetermined concentration of allergen in a closed, controlled environment and provide a mechanism to induce clinical symptoms and measure the effect of medication.

## Methods

A preliminary evaluation of the capabilities of the newly constructed Red Maple Trials Allergen Challenge Theatre^TM^ was performed. Health Canada and a provincial Ethics Board approved the study. After signing informed consent, patients with a history of grass allergy, not on allergy medications and with a positive skin prick test to grass antigen (≥ 3 mm) were exposed for 3 hours to timothy grass pollen (*Phleum pratense*) in the allergen challenge theatre. Total nasal (TNSS) and rhinoconjunctivitis symptom scores (TRSS) were recorded at baseline and every 30 minutes during the challenge.

## Results

32/50 patients evaluated demonstrated a positive skin prick test and were challenged. Baseline TNSS and TRSS (Mean± SD) were 0.6±1.04 and 0.6±1.07 respectively. Symptom scores reached a plateau at 30 minutes (TNSS 4.8±2.68; TRSS 5.8±3.69) and remained steady for the 180-minute exposure period reaching final values of TNSS 3.7±2.16; and TRSS 5.8±3.79. Because entry to a therapeutic trial usually requires achieving a TNSS ±5 during a priming exposure, we calculated the results for the 17/32 patients reaching this score at 30 minutes (TNSS 6.65±2.21; TRSS 8.35±3.18). Scores held steady and at 180 minutes were: TNSS 4.71±1.69; TRSS 7.88±3.06. No unexpected adverse events were reported during the challenge.

## Conclusions

The Red Maple Trials allergen exposure theatre demonstrated the capacity to induce symptoms of appropriate intensity upon allergen challenge. The chamber with a seating capacity of 99 places has the ability to evaluate large test groups at a time.

